# Development of Odour-Baited Flytraps for Sampling the African Latrine Fly, *Chrysomya putoria*, a Putative Vector of Enteric Diseases

**DOI:** 10.1371/journal.pone.0050505

**Published:** 2012-11-30

**Authors:** Thomas C. Lindsay, Musa Jawara, Umberto D’Alessandro, Margaret Pinder, Steven W. Lindsay

**Affiliations:** 1 Department of Disease Control, London School of Hygiene and Tropical Medicine, London, United Kingdom; 2 Disease Control and Elimination Theme, Medical Research Council Unit, Fajara, The Gambia; 3 Unit of Malariology, Institute of Tropical Medicine, Antwerp, Belgium; 4 School of Biological and Biomedical Sciences, Durham University, Durham, United Kingdom; University of Crete, Greece

## Abstract

African pit latrines produce prodigious numbers of the latrine fly, *Chrysomya putoria*, a putative vector of diarrhoeal pathogens. We set out to develop a simple, low-cost odour-baited trap for collecting *C. putoria* in the field. A series of field experiments was carried out in The Gambia to assess the catching-efficiency of different trap designs. The basic trap was a transparent 3L polypropylene box baited with 50 g of fish, with a white opaque lid with circular entrance holes. We tested variations of the number, diameter, position and shape of the entrance holes, the height of the trap above ground, degree of transparency of the box, its shape, volume, colour, and the attractiveness of gridded surfaces on or under the trap. Traps were rotated between positions on different sampling occasions using a Latin Square design. The optimal trapping features were incorporated into a final trap that was tested against commercially available traps. Features of the trap that increased the number of flies caught included: larger entrance holes (compared with smaller ones, p<0.001), using conical collars inside the holes (compared with without collars, p = 0.01), entrance holes on the top of the trap (compared with the side or bottom, p<0.001), traps placed on the ground (compared with above ground, p<0.001), the box having transparent sides (compared with being opaque, p<0.001), and with no wire grids nearby (compared with those with grids, p = 0.03). This trap collected similar numbers of *C. putoria* to other common traps for blow flies. The optimum trap design was a transparent box, with a white plastic lid on top, perforated with 10 conical entrance holes, placed on the ground. Our simple trap provides a cheap, low-maintenance and effective method of sampling *C. putoria* in the field.

## Introduction

Diarrhoea is responsible for killing about 1.5 million children each year [1]. An important route of infection is thought to be the mechanical transmission of diarrhoeal pathogens by flies [2]–[4]. *Chrysomya putoria*, the African latrine fly, may be a possible vector of enteric infections since they are strongly attracted to human faeces, harbour faecal pathogens and feed on raw meat and fish in large numbers [5]. It is likely that such bacterial pathogens on the food are spread when people handle the contaminated food, eventually infecting themselves and possibly others.

There have been few surveillance studies of *C. putoria* in sub-Saharan Africa and there are no traps specifically developed for sampling this fly. Since *C. putoria* are ubiquitous in many rural settings [5]–[7] we set out to explore how best to trap these flies, since a trap may be used for surveillance purposes or, if highly effective at trapping flies, as a possible control tool.

Fly traps often exploit two common fly behaviours, an attraction to an odour source and bright light [8]. The odours lure the flies into the trap while the sunlight misdirects their exit path so that they remain within the trap. Since *Chrysomya* spp. are blowflies, traps are often baited with bullock’s liver [9] or raw fish [10]. There are numerous features of a trap which may affect its catching efficiency including the number, diameter, position and shape of the entrance holes, the height of the trap above ground, degree of transparency of the box, its shape, volume, colour and the attractiveness of gridded surfaces near the trap [8]. The entrance holes must be large enough and numerous to allow flies to readily enter the trap, but not too large or numerous for them to exit freely. The position of the entrance holes on the trap can also affect trapping with some fly species preferring to enter traps from the top [11], bottom [12] or sides [13]. The shape of the entrance hole is important too. Entrance holes with conical collars, tubular tunnels or baffles projecting into the trap chamber may restrict flies leaving the trap by blocking direct line of sight to the exit [8], [13], [14]. Flytraps positioned on the ground may outperform those that are elevated [10]. Two explanations for this are flies are forced lower to the ground to avoid high wind speeds [15] and a fly’s natural feeding and breeding media are often close to or on the ground. However, this is not always the case since one study found that traps with entrance holes 25.4–45.8 cm above the ground performed best [8]. Flies are known to be drawn to light sources (positively phototaxic), so on entering the trap they move towards the brightest light source, so the degree of transparency of the trap may affect trap catch size. The shape of the trap can affect sampling with rectangular traps considered better than cylindrical ones [8]. Volume may be important since a trap that is too small will lose flies from the containment area, whilst one that is too big is not cost effective. Flies are attracted to mainly white, yellow or blue [8], [11] while black can be repellent [11]. Flies prefer to land on the edge of objects where the contrast in colour is greatest [16], a behavioral characteristic used in the design of the Scudder grill; a device to estimate house fly populations [17]. We tested whether we could increase catch size by laying wire grids on the lid of the trap or resting a trap on a grid. We conducted a series of experiments testing each of these features separately to optimize trap design.

We tested this trap against a number of common traps used for sampling flies. These included (1) the baited-cone trap [12], (2) Emerson and co-workers trap [18] (3) the LuciTrap (Bioglobal Ltd, Eight Mile Plains Australia) and (4) the Agrilure (Agrimin Ltd, Brigg UK). The purpose of this series of experiments was to develop a cheap and simple trap for collecting *C. putoria* that was as good as or better at catching flies than other common flytraps, and could be installed on pit latrines, where these flies are attracted to and emerge from.

## Materials and Methods

### Ethical Statement

Ethical approval for this study was provided by The Gambia Government/MRC Laboratories Joint Ethics Committee as well as the London School of Hygiene and Tropical Medicine’s Ethics Committee. No specific permit was required for fly trapping at the MRC Unit’s field station at Basse since approval had already been given by the local institutional review board. Verbal consent for fly trapping was provided by household heads in Kundam Demba village. The field studies did not involve endangered or protected species.

### Study Sites

The trap development studies were carried out inside and close to the Medical Research Council’s (MRC) field station on the outskirts of Basse Santa Su (13°18′37.48′′N, 14°13′24.46′′W), a rural town in the Upper River Region of The Gambia. Trap comparisons were performed in Kundam Demba village (13°20′13.51′′N, 14°7′3.43′′W) between June and December in 2011. This is an area of open Sudanian savannah with a rainy season from May to October followed by a long dry season. Most people live in small rural villages in houses with mud or cement walls and thatched or metal roofs. Houses are mainly grouped in compounds and toilets are usually pit latrines shared by several members of a compound, although open defaecation also occurs. The trap development site close to the MRC was a communal open-defaecation area used by local farmers.

### Study Design

Experiments were based on a Latin Square design that is used to adjust for variation in fly numbers due to trap, position and day. If a design feature significantly increased the catch sizes, it was incorporated into the basic fly trap design and tested in successive experiments. Traps were positioned in a straight line, 2 m apart, and fly collections made between 09∶00 and 17∶00 h, when fly numbers were greatest. Collections were normally made after 4 hours, but were extended to 6 hours when fly numbers were low in order to stabilise the variance. Flies were killed by freezing at −20°C for 2 hours, identified to species and sex and then counted.

A pilot 6×6 Latin Square comparison study was carried out, with three treatments duplicated, to estimate the variation in fly numbers between traps on different days. The mean natural logarithm of total *C. putoria* collected in each trap was 1.5 (SD = 1.4) for the small holes, 2.7 (SD = 1.5) for the medium holes and 3.4 (SD = 1.3) for the large holes. Fly counts were log transformed to stabilise the variance. Since the large holes caught most flies, this became our reference trap. We were interested in detecting only large differences in catching efficiency between different trap designs, so we designed our experiments to detect a 50% increase or decrease in fly numbers relative to the reference trap, at the 5% level of significance and with 80% power. Using the information above in a web-based sample size calculator (http://stat.ubc.ca/~rollin/stats/ssize/n2.html, accessed June 2011) we required a sample size of 10. Thus we adopted a 6×6 Latin Square design with repeats of each trap on each trapping occasion, so that at the end of each experiment each trap design was tested 12 times (i.e. 2×6 days). Treatments A to E in the Latin Square were randomly allocated a number from 1 to 6 for each experiment. Flytraps were then randomly allocated to one of these numbers. The Latin Square allowed each treatment to be allocated a different position each day, so that at the end of each experiment each trap had been in each position and was never continuously next to the same neighbouring trap. Thus, at the end of each experiment the number of flies caught in each trap type will be independent of their neighbour.

### Trap Development

The basic trap used to collect flies was a 3L volume (17 cm^3^), transparent polypropylene box with a snap-top white opaque lid (Whitefurze, Coventry UK), perforated with 10, 1.6 cm diameter holes. The bait was 50 g of raw common catfish, *Synodontis batensoda*, since this was shown to be attractive to *C. putoria* in earlier studies [5]. The fish was placed in a white plastic pot 250 cm^3^ in volume (6 cm in height and 9 cm diameter; W. K. Thomas, Chessington UK), covered with a cotton-netting lid, secured by an elastic band and placed in the centre of the floor of the trap.

Preliminary experiments used traps with adhesives, but they were relatively ineffective at trapping large, strong flies, so this was abandoned. We also tested traps painted entirely in blue, white or brown paint, but these caught very few flies. Each experiment tested two or three variations of the basic trap including the diameter, number, position and shape of the entrance holes, whether or not the trap was left out overnight, the height above ground, degree of transparency of the trap walls, shape of trap (cubic or cylindrical), volume, colour and the attractiveness of gridded surfaces on the trap. The precise details of these experiments and the rationale for testing them is summarised in table 1.

**Table 1:** Experiments for developing a flytrap for collecting *C. putoria*.

**Trap feature**	**Variables tested** a	**Rationale for experiment**
Entrance hole - diameter	0.6 cm, 1.0 cm and 1.6 cm diameter entrance holes.	To determine the ideal size of hole for collecting and holding flies in a trap.
Entrance hole - diameter and number of holes	28×0.6 cm diameter entrance holes, 10×1.0 cm diameter entrance holes and 4×diameterentrance holes.	Here the total area of hole is the same for the small, medium and large holes. We hypothesized that if hole size was unimportant, all traps would collect a similar number of flies.
Entrance hole - position	Top, bottom and side, with all holes positioned17 cm above the ground	To determine whether fly catch size was dependent on whether the entrance holes were on the top, bottom or side of the trap
Entrance hole – shape test 1	Standard 1.6 cm diameter entrance holes, (1) without a cone fitted, (2) with a 1.0 cm deep paper cone with a 0.6 cm internal diameter at its tip, (3) with a 1.0 cm cone with a 1.0 cm internal diameter at its tip.	We hypothesized that small conical collars protruding from the entrance holes inside the trap lid would reduce the number of flies leaving a trap.
Entrance hole – shape test 2	Standard 1.6 cm diameter entrance holes, fittedwith and without a 0.6 cm diameter paper cone,1.0 cm deep. Traps were left overnight.	Although the 1.6 cm holes were effective at letting flies enter traps, we hypothesised that conical entrance holes would make it less likely for flies to leave the traps at night.
Trap - height	Bottom of trap positioned 0 cm, 25 cm and 120 cm above the ground.	To determine whether the height of a trap affected fly collections.
Trap - opacity	Transparent (no paper), semi-transparent (1 layer of 90 gm^2^ Natural Translucent Paper (Royal SovereignLtd., London, UK)) and opaque (3 layers of 90 gm^2^Natural Translucent Paper). The paper waspositioned inside the traps.	We hypothesized that flies remained in the trap since they were attracted to the light coming through the transparent walls. We considered that non-transparent flies would have fewer flies.
Trap - shape	Cubic 1L box (Whitefurze, Coventry UK) and acylindrical 1L box (Whitefurze, Coventry UK).	We hypothesized that flies were more likely to leave a trap which had internal corners than one which did not.
Trap - volume	0.6 L, 3.0 L and 6.0 L boxes	To determine whether the trap volume affected fly collections.
Trap – lid colour only	The plastic lids were replaced with either glosswhite, brown or blue mount board (Antique White,Chocolate Murano, Hussar Blue, Daler-Rowney,Bracknell UK) cut to fit. Entrance holes wereadded to the lid of each trap.	To determine whether the colour of the lid affected fly collections. White was the standard lid colour, brown is the colour of faeces and blue is a common colour used for attracting tsetse flies, a day-flying insect.
Trap – slab colour	Grey 1×1 m chipboard, black 1×1 m chipboard and blue 1×1 m chipboard. A 12 cm diameter pipe,10 cm in length, in the centre of each board wasinserted into the base of a standard trap	During the ‘Trap – lid colour only’ experiment we noticed flies were least attracted to black lids. We tested whether colours under the trap would deter flies from entering the trap.
Trap - gridded vs. non-gridded	1×1 m wire grid placed underneath the trap, 0.17×0.17 m wire grid positioned over the traplid, no grid. The wire grid consisted of 1 mm steelwire spaced 2.5 cm apart.	Based on the characteristics of a Scudder Grill. A grid is recognized as a means of counting flies in a given area because they (the flies) prefer to land on edges.

a Standard trap design consisted of 10 entrance holes, each 1.6 cm in diameter, in a white lid of a 3 L polypropylene box, with transparent sides. 50 g of raw fish was placed in a 9 cm diameter white pot, covered with cotton netting.

### Trap Comparisons

**Figure 1:**
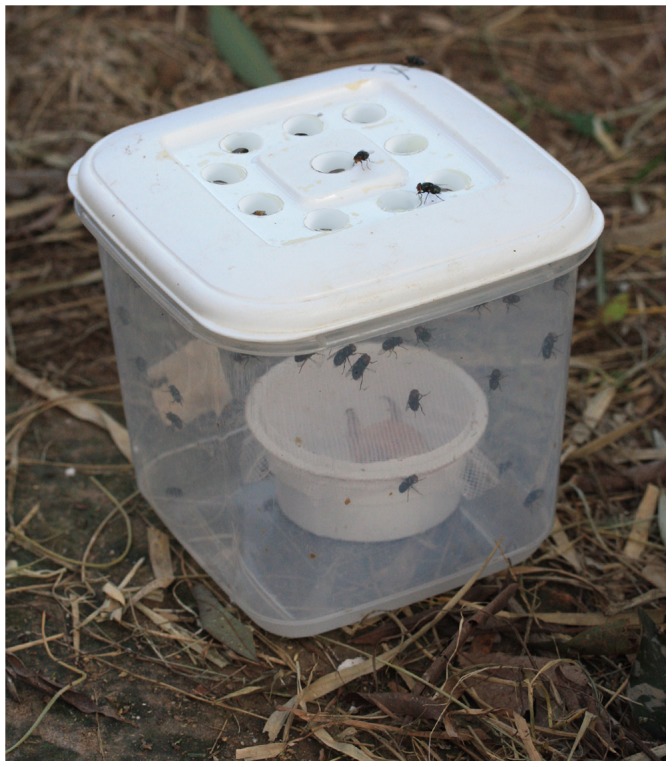
Optimal trap for collecting *C. putoria*.

**Figure 2:**
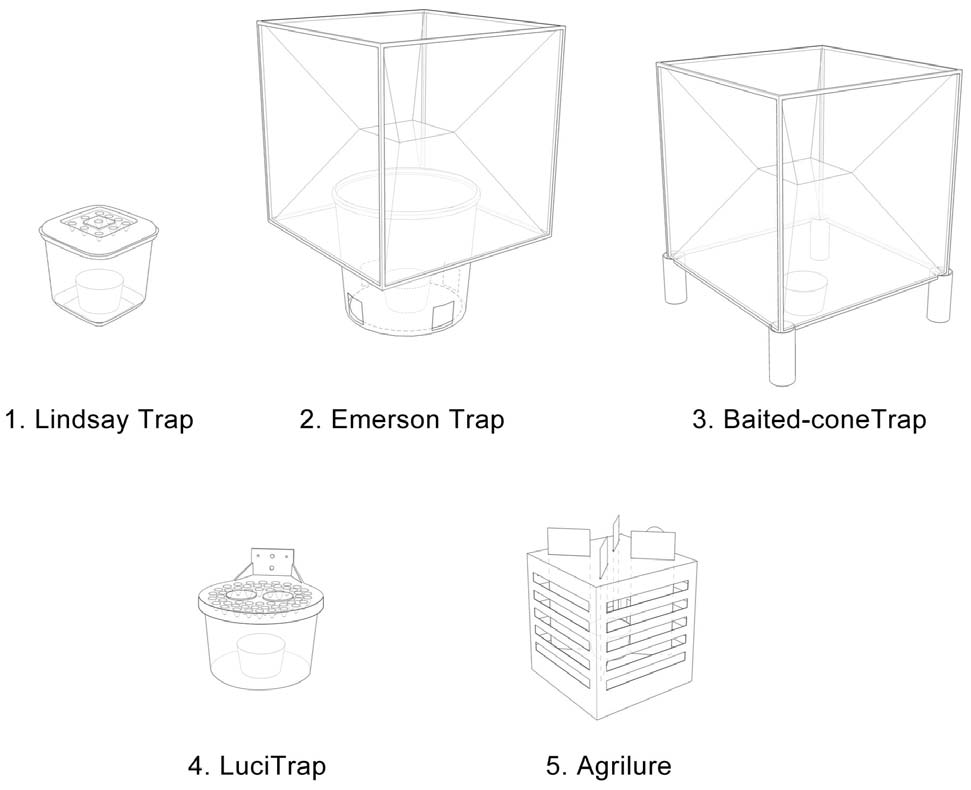
Common flytraps used for collecting blow flies.

Our final trap consisted of 10, 1.6 cm diameter entrance holes in a white snap-top opaque lid of a 3 L polypropylene box, with transparent sides (Figure 1). The entrance holes had conical collars inserted in the trap each 1 cm in length and 0.6 cm in diameter at the smallest opening and pointing into the trap chamber. We tested this trap against several other traps (Figure 2) used for collecting flies including (1) the baited-cone trap [12], (2) Emerson and co-workers trap [18], (3) Agrilure (Agrimin Ltd, Brigg UK) and (4) LuciTrap (Bioglobal Ltd, Eight Mile Plains Australia). Baited-cone traps were 40 cm^3^ mosquito exit traps with the entrance 10 cm above the ground, supported by 5 cm diameter grey plastic piping at each corner. Emerson traps were 40 cm^3^ mosquito exit traps with the entrance pointed down into blue plastic buckets (diameter 24 cm, 20 cm at the base) which had three 5 cm^2^ holes on the sides near the base. Agrilure traps were 30 cm^3^ white corrugated-plastic cubes with horizontal entrance slits and within the trap were four vertical black adhesive strips to trap the flies. LuciTraps were UV stabilised, semi-transparent plastic buckets with flat yellow lids, 23.1 cm in diameter, perforated with 50 conical entrance holes, 1.5 cm diameter on entry, 1.5 cm deep and 0.5 cm diameter on exit. All traps were baited using 50 g of fish placed as indicated above. This final experiment used a 10×10 Latin Square design since there were 5 trap designs, two of each, to test.

### Statistical Analyses

Fly counts were transformed using natural logarithms to normalize the data. General linear modelling was used to account for the variation in fly numbers between different trap designs, position of trap, day and replicate. Comparisons of traps within each experiment were made using Bonferonni Statistical analysis using SPSS version 19.0.

## Results

In total, the traps caught 9,200 flies: 52.6% were *Chrysomya putoria* (n = 4,840), 25.4% were *Chrysomya marginalis* (n = 2,336), 8.1% were *Musca* spp. (n = 745), 7.2% were *Lucilia cuprina* (n = 663), 5.4% were *Sarcophaga* spp. (n = 497) and 1.3% were classed as ‘other’ species (n = 119).

**Figure 3:**
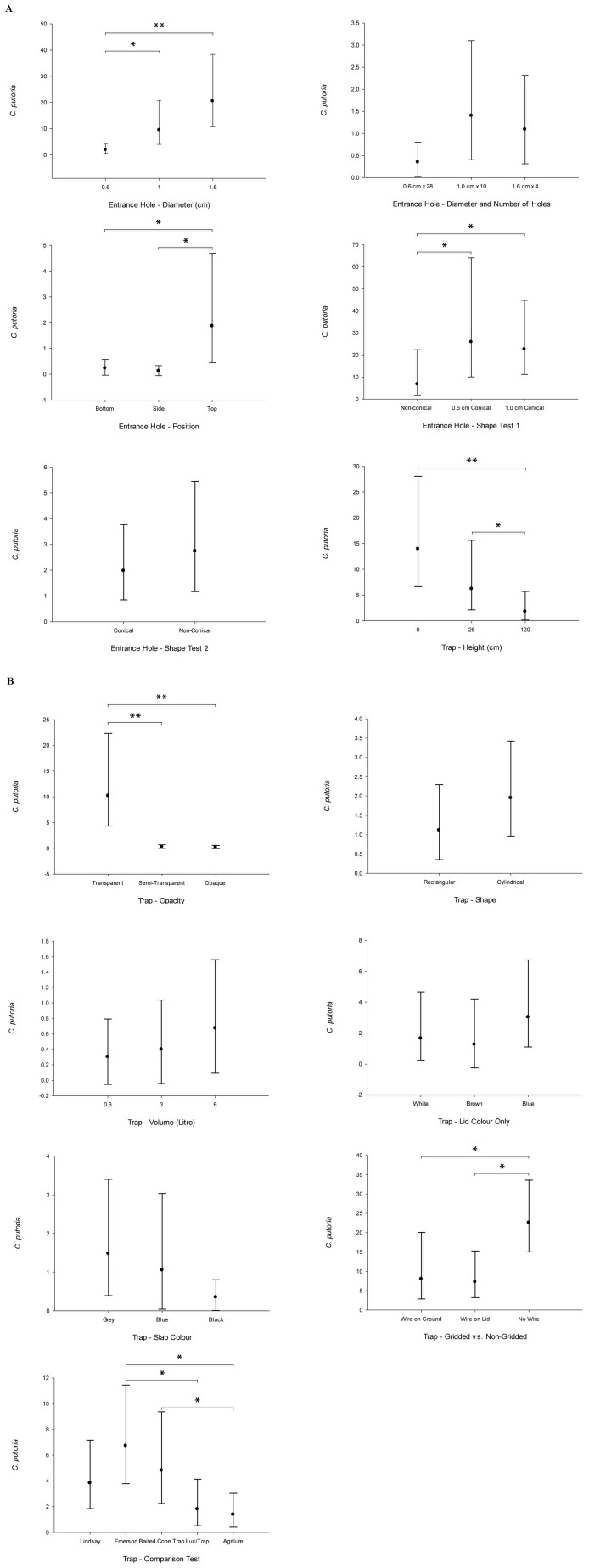
a & b. Trap development experiments. Means and 95% confidence intervals are shown, where * = P<0.05, ** = P<0.001).

Several features of the trap influenced the number of flies collected (Figure 3a and 3b) including; the entrance hole size (F = 34.70, df = 2, p<0.001), whether the entrance holes had conical collars of not (F = 9.39, df = 2, p = 0.01), the position of the entry holes (F = 9.74, df = 2, p<0.001), the height of the trap (F = 26.38, df = 2, p<0.001), the opacity of the walls (F = 34.26, df = 2, p<0.001) and the presence or absence of a gridded surface (F = 7.47, df = 2, p = 0.03). Some features did not alter the trap catch size, including changing the diameter of the entrance holes, whilst adjusting the number of holes, so that the total area of entrance holes was the same for each trap (F = 3.25, df = 2, p = 0.06), if traps had cones or not and were left overnight (F = 0.843, df = 2, p = 0.37), the shape of the trap (F = 3.16, df = 2, p = 0.09), the volume of the trap (F = 1.55, df = 2, p = 0.23), the lid colour (F = 1.45, df = 2, p = 0.26) and slab colour (F = 1.36, df = 2, p = 0.28). Details of each trap feature tested are provided in table 1.

Although larger holes caught more flies than smaller holes, the catch size to surface area ratio for 0.6 cm diameter holes (1∶13) was similar to the ratio for 1.6 cm diameter holes (1∶18). When we adjusted the number of holes in the lid so that the surface area of entry holes was similar, we found no significant difference between catch size, confirming our hypothesis that hole size diameter between 0.6 and 1.6 cm is not a critical feature of the trap, rather it is the total surface area of holes that is important.

Whilst the number of flies collected differed significantly between the traps (F = 5.321, df = 4, p = 0.001), our trap was not significantly better than the Emerson, baited-cone trap, LuciTrap and Agrilure (p = 1.000, 1.000, 1.000, 0.583 respectively). In terms of cost per unit, the LuciTrap cost $31.53, the Agrilure cost $15.63, the Emerson Trap cost $9.66, the baited-cone trap cost $8.80 and our box trap cost $3.92. Both the LuciTrap and Agrilure included bait in the cost of the trap.

## Discussion

We developed a simple and cheap fly trap for collecting *C. putoria* by incorporating the most effective design features identified from a series of simple experiments. Raw fish as a bait was used successfully for collecting *C. putoria* in a range of different traps, although occasionally local cats looking for food upturned the traps. Fish-baited traps have also been used successfully for collecting *Chrysomya* spp. in other studies in The Gambia [5], [10].

Fly catch size increased as the entrance hole size increased from 0.6 cm to 1.6 cm diameter. However, since we used the same number of holes in each trap, the total hole area also increased with hole size. When we controlled for this by varying the number and size of hole, while maintaining an equal hole area, in this case we found that each trap variant collected similar numbers of flies. Thus the size of individual holes is less important than total hole area. Of the three individual hole sizes tested, we found holes 1.6 cm in diameter collected most flies. The position of the entrance holes on the trap was important, with highest fly catches obtained when the holes were on the top of the trap, compared with the side or bottom. This finding is probably a result of two factors. Firstly, since flies search for food or an oviposition medium by flying low to the ground this is the nearest entrance for the fly, although visual cues may also be important. Secondly, the attractive odours that are released from the fish within the trap may be more readily located when the odours rise from the top of the trap, than the side or bottom. Many fly traps reduce the exit rate of flies by having a single large entrance or numerous smaller entrances, each with a conical collar pointing into the trap. Traps fitted with conical collar entrance holes collected more flies than those without collars during the day, but there was no difference when left overnight. Natural light serves as the secondary lure in our traps. At night, the light attracting flies to the sides of the traps reduced in intensity, and many flies left the trap. Our strong recommendation is that these fly traps should not be left out overnight as many flies will be lost from the catches.

Fly catches were greatest in traps placed on the ground with entrance holes on top of the box. Similarly, Emerson found fish-baited traps left on the ground caught more flies than those suspended above the ground [10]. It is likely that this results from the behaviour of the blowfly when seeking faeces or raw meat, both of which are found at ground level with the largest available landing surface facing upwards. Furthermore, traps positioned above the ground are subject to stronger winds that may disturb the trap, making it less attractive as a landing platform for these highly sensitive flies, which are sensitive to movement when locating a breeding or feeding medium.

Daylight is an important factor influencing fly catches. Once inside the trap the flies were attracted to fish bait, but since this is covered by netting, we assume that many will attempt to feed or oviposit elsewhere. When inside the trap the flies are attracted to light and the greatest light source in the trap are the transparent sides. Hence we were able to demonstrate that as the walls of the trap became more opaque they caught fewer flies, since the predominant light source in these traps were the holes in the lid of the trap, from which they exited.

Whilst more flies were caught in cylindrical traps than rectangular ones this result was not statistically significant. We had hypothesised that the corners of the traps may cause flies to fly off the surface, unlike in cylindrical traps where the flies continue to circle around the trap. Catch size increased with the volume of the trap, but this trend was not significant. Here we hypothesised that in larger traps flies would be less likely to collide with one another, eliciting take off and hence exit from the trap, as well as the flies being less likely to find an exit from the trap. Nonetheless smaller traps would be preferred since they are cheaper than larger ones.

There was no significant difference between the catch sizes of traps with different coloured lids nor the colour of the base on which the trap was positioned. However, fewer flies were collected in the traps when positioned on a black base. The lack of statistical significance in this experiment may be due to the very low fly numbers collected.

Whilst it is well known that flies are attracted to edges and corners [19], wire grills placed over or under the trap reduced the number of flies collected in the trap. Presumably flies landed on the grids in preference to resting on the traps.

When we compared a range of different traps used commonly for collecting flies our trap performed as well as the others. Overall the Emerson and baited-cone trap performed best when compared with the LuciTrap and Agrilure, yet there are practical reasons for not using the Emerson and baited-cone trap on latrines. The netted sides make it impractical for long-term use on a latrine. A study in Dar es Salaam found even wire screens attached to latrine vents had degraded after a year [20]. The LuciTrap and Agrilure were surprisingly ineffective for traps designed to catch blowflies. Although marketed as such, their design seems unsuited for catching *C. putoria*. The Agrilure has adhesive strips that failed to trap the relatively powerful blowfly. Additionally, the sticky strips lost their adhesiveness over time, which makes them too high maintenance for our needs. A cost comparison showed that our box trap was considerably cheaper than the other traps, although it is unfair to compare the price of our trap with the cost of the LuciTrap and Agrilure since these are marketed for profit and their price does not reflect the true construction costs. They also both include not only a fly trap but the bait as well in the price. Both the Emerson and baited-cone trap were made while in Basse using locally brought materials and locally made and therefore reflect only the base construction costs. A cost we did not deliberately factor is the bait, a commodity that must always be renewed regardless of trap design.

The box trap was a cheap and effective method for collecting *C. putoria* and could be used for routine surveillance and for collecting flies from latrines.
